# Organokines in COVID-19: A Systematic Review

**DOI:** 10.3390/cells12101349

**Published:** 2023-05-09

**Authors:** Sandra Maria Barbalho, Giulia Minniti, Vitor Fernando Bordin Miola, Jesselina Francisco dos Santos Haber, Patrícia Cincotto dos Santos Bueno, Luiza Santos de Argollo Haber, Raul S. J. Girio, Cláudia Rucco Penteado Detregiachi, Camila Tiveron Dall’Antonia, Victória Dogani Rodrigues, Claudia C. T. Nicolau, Virginia Maria Cavallari Strozze Catharin, Adriano Cressoni Araújo, Lucas Fornari Laurindo

**Affiliations:** 1 Department of Biochemistry and Pharmacology, School of Medicine, University of Marília (UNIMAR), Avenida Hygino Muzzy Filho, 1001, Marília 17525-902, SP, Brazil; 2 Postgraduate Program in Structural and Functional Interactions in Rehabilitation, University of Marília (UNIMAR), Avenida Hygino Muzzy Filho, 1001, Marília 17525-902, SP, Brazil; 3 Department of Biochemistry and Nutrition, School of Food and Technology of Marília (FATEC), Avenida Castro Alves, 62, Marília 17500-000, SP, Brazil; 4 Centro Interdisciplinar em Diabetes (CENID), School of Medicine, University of Marília (UNIMAR), Avenida Hygino Muzzy Filho, 1001, Marília 17525-902, SP, Brazil; 5 Department of Animal Sciences, School of Veterinary Medicine, University of Marília (UNIMAR), Avenida Hygino Muzzy Filho, 1001, Marília 17525-902, SP, Brazil; 6 Department of Biochemistry and Pharmacology, Faculdade de Medicina de Marília (FAMEMA), School of Medicine, Avenida Monte Carmelo, 800, Marília 17519-030, SP, Brazil

**Keywords:** SARS-CoV-2, COVID-19, organokines, adipokines, myokines, osteokines, hepatokines, cardiokines, viral infection, pandemic, immune system

## Abstract

Coronavirus disease 2019 (COVID-19) is a viral infection caused by SARS-CoV-2 that induces a generalized inflammatory state. Organokines (adipokines, osteokines, myokines, hepatokines, and cardiokines) can produce beneficial or harmful effects in this condition. This study aimed to systematically review the role of organokines on COVID-19. PubMed, Embase, Google Scholar, and Cochrane databases were searched, the Preferred Reporting Items for Systematic Reviews and Meta-Analyses (PRISMA) guidelines were followed, and 37 studies were selected, comprising more than 2700 individuals infected with the virus. Among COVID-19 patients, organokines have been associated with endothelial dysfunction and multiple organ failure due to augmented cytokines and increased SARS-CoV-2 viremia. Changes in the pattern of organokines secretion can directly or indirectly contribute to aggravating the infection, promoting immune response alterations, and predicting the disease progression. These molecules have the potential to be used as adjuvant biomarkers to predict the severity of the illness and severe outcomes.

## 1. Introduction

During the last two decades, coronaviruses have spread thrice worldwide and caused life-threatening diseases among humans from all continents. Besides the severe acute respiratory syndrome coronavirus (SARS-CoV) and the Middle East respiratory syndrome coronavirus (MERS-CoV), the new severe acute respiratory syndrome coronavirus 2 (SARS-CoV-2) emerged in China in December 2019 and has been responsible for a pandemic since then, leading to millions of deaths. Although vaccines were developed against this pathogen and most of the world’s population has been vaccinated, this virus continues to circulate and evolve, and variations with new proteins arise [1,2,3]. Most infected people develop mild symptoms, but many health conditions have been associated with coronavirus disease 2019 (COVID-19), such as dyspnea, myalgia, cognitive impairment, and amnesia [4,5]. SARS-CoV-2 is a positive-sense single-stranded RNA genome virus and shares 79% of its genome sequence identity with SARS-CoV and MERS-CoV. This RNA genome is packed in a spherical envelope (E) with specific surface glycoprotein projections called spikes. Besides the spike (S) and the E proteins, SARS-CoV-2 also presents two other unique protein structures called the membrane (M) and the nucleocapsid (N). Pathogenically, this viral infection initiates with the binding of the SARS-CoV-2 S protein on the host cellular superficial receptors. The S protein interacts with the angiotensin-converting enzyme 2 (ACE2) receptors of the host cells, and the type II transmembrane serine protease 2 (TMPRSS2) activates the viral entry. After this process, SARS-CoV-2 starts to multiply its genome, and at the end of the replication, it assembles and leaves from the primary-infected cells to infect other susceptible cells [6,7,8]. 

The condition resulting from the SARS-CoV-2, COVID-19, is primarily considered a systemic pro-inflammatory illness that induces oxidative stress (OS). The cytokine storm is an uncontrolled hyper-inflammation that occurs during COVID-19, leading to acute respiratory distress syndrome (ARDS) and multiple organ failure. COVID-19 is also related to an imbalance in immunological cells, principally in mild-to-severe cases where there is a reduction in the bloodstream T and B cells [9,10,11].

Considering that COVID-19 is a severe inflammatory disease characterized by the enormous production of pro-inflammatory cytokines, the study of organokines (adipokines, myokines, osteokines, hepatokines, and cardiokines) in this disease is needed. These molecules are produced mainly by the adipose tissue (AT), skeletal muscles, bones, liver, and heart, respectively. They are associated with many inflammatory conditions, such as rheumatoid arthritis, non-alcoholic fatty liver disease, dementia, and cardiovascular affection. Since organokines act through autocrine, paracrine, or endocrine pathways, they can play a potential role in the COVID-19 pathophysiology [12,13,14].

Organokines are involved with the burden of inflammation, development of cardiorenal injuries, disease progression, control of symptoms and disease severity, lung tissue repair, immunological regulation or dysregulation, and SARS-CoV-2-mediated acute sarcopenia in COVID-19 [15,16,17,18]. Some studies have shown that organokines are highly associated with adipocyte dysfunction in this condition. Moreover, they can be related to endothelial injuries, leading to significant disease severity. Higher levels of specific organokines were also associated with multiple organ failure, such as encephalitis [19,20,21,22].

Considering that organokines can act individually or through a cross-talk between different organs to improve health or cause disease [23] and are associated with COVID-19 disease and progression, this study aimed to conduct a systematic review of adipokines, myokines, osteokines, hepatokines, and cardiokines and their roles in COVID-19. To the best of our knowledge, this is the first review considering the actions of organokines in COVID-19. We hope this study will contribute to developing new and modern diagnostic and therapeutic strategies for the healthcare of people infected by SARS-CoV-2.

## 2. Materials and Methods

### 2.1. Focal Question

The focal question considered in this review was “What is the role of adipokines, myokines, osteokines, hepatokines, and cardiokines on the pathophysiology of COVID-19”?

### 2.2. Language

We included in this review only studies published in English.

### 2.3. Databases

We built this review using PubMed, Embase, Google Scholar, and Cochrane. The mesh terms used were “organokines” and “adipokines” and “myokines” and “osteokines” and “hepatokines” and “cardiokines” combined with “COVID-19” and “SARS-CoV-2”. The mesh terms enabled the search and identification of clinical studies and other studies related to this systematic review’s objectives. We used the Preferred Reporting Items for Systematic Reviews and Meta-Analyses (PRISMA) flow diagram to perform a more accurate search for studies related to organokines and COVID-19 [24].

### 2.4. Study Selection

The inclusion criteria comprised retrospective observational case-control studies; retrospective cross-sectional studies; prospective cohort studies; pilot cohort studies; single-center, prospective observational studies; single-center cohort, cross-sectional studies; autopsy studies; comparative, observational studies; and prospective studies. Only full texts were considered.

The exclusion criteria comprised animal models, in vitro studies, studies published in languages other than English, reviews, poster presentations, case reports, and editorials. Descriptive and systematic reviews helped to build the discussion section of this study.

### 2.5. Data Extraction

Only studies published from 1st January 2020, to 1st January 2023, were selected to compose this review. Given that the World Health Organization (WHO) declared the pandemic in March 2020, we consider this period adequate.

## 3. Discussion

### 3.1. Pathophysiology of the COVID-19 Infection

SARS-CoV-2 is transmitted via aerosols and droplets from person to person. This is considerably large for being 60 nm to 140 nm in diameter and having an envelope and a nucleocapsid forming its structure. It is a single-stranded positive RNA virus, and its virion is approximately 50 to 200 nm in diameter, exposing four distinct structural proteins: S, E, and M, which participate in the formation of the viral envelope, and an N, the protein that holds the unique positive RNA genome of the virus. The S protein is composed of two functional subunits, S1 and S2, among which S1 is responsible for binding to the host cell receptor, and S2 plays a role in the fusion of viruses in host cell membranes. Therefore, it is essential for attachment and penetration into the host [25,26,27]. 

The primary infection targets of this virus are nasal and bronchial epithelial cells, in addition to pneumocytes of the pulmonary respiratory epithelium. The process of host cell invasion occurs through the binding of protein S to a functional receptor for SARS-CoV-2, the ACE2, highly expressed in type 2 pneumocytes of the pulmonary respiratory epithelium. After binding of SARS-CoV-2 to ACE2, the S protein undergoes an activation stimulus through cleavage by the TMPRSS2. Once inside these cells, SARS-CoV-2 can provoke an initial immune response. The virus-mediated immune response occurs mainly by the release of inflammatory cytokines in addition to a weak interferon (IFN) response, which can amplify the inflammatory reactions and lead to an amplification of the cytokine storm [25,28]. 

The process of invasion occurs in two steps. The first is for priming at the S1 cleavage site and S2, and the second cleavage is for activation at a position adjacent to a fusion peptide within the S2 subunit. In summary, the initial partition stabilizes the S2 subunit at the attachment site. The subsequent division activates the S protein causing conformational changes that lead to the fusion of the viral membrane and the host cell [29,30].

After the transcription process, newly formed nucleocapsids are encased in the endoplasmic reticulum membrane and transported to the lumen, where transport occurs via Golgi vesicles to the cell membrane. Then, genomic RNA is incorporated as the particles mature via budding exocytosis into the extracellular space. Thus, new viral particles are released, invading adjacent epithelial cells and providing new infectious material for community transmission [25,26,27]. 

**Figure 1:**
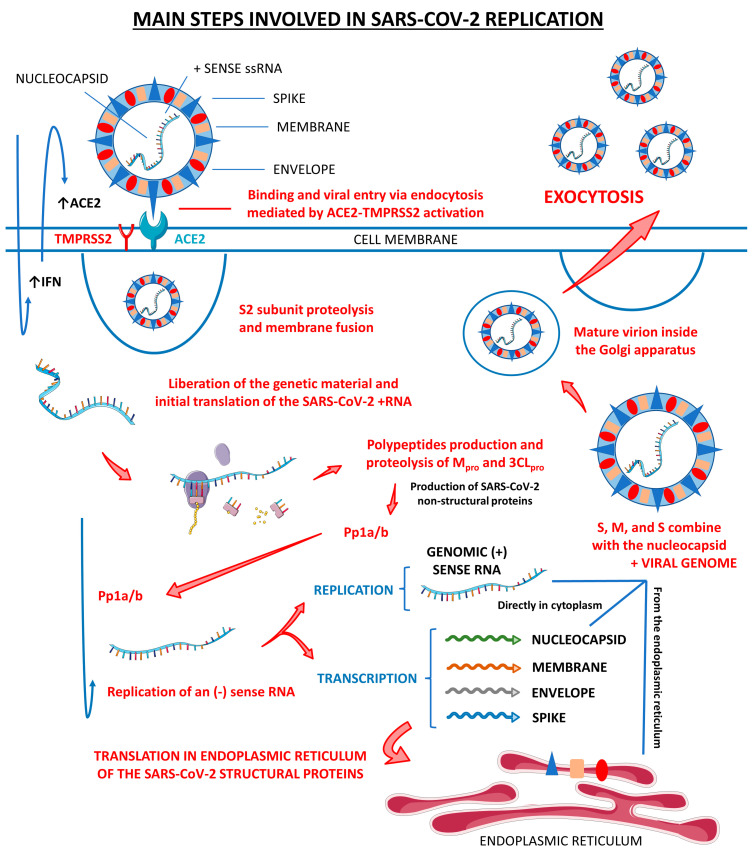
Main steps involved in SARS-CoV-2 replication. There are three host cells in which SARS-CoV-2 can replicate most effectively: nasal goblet epithelial cells, type 2 pneumocytes, and enterocytes. This virus enters the human organism through the nasal–oral route upon exposure. In response to this aggression, the body starts to respond by the activation of the innate immune system, which initiates producing interferon (IFN). These IFN molecules interact with the angiotensin-converting enzyme 2 (ACE2, the receptor for the SARS-CoV-2 attachment to the host cell) and activate it, opening the cells’ doors to the virus entrance. Mediated by the type II transmembrane serine protease 2 (TMPRSS2), SARS-CoV-2 fusions its membrane to the host cells and liberates its genome. The virus uses the host cells’ machinery to translate its (+) sense RNA genome into non-structural proteins, which are the main proteases M^pro^ or 3CL^pro^, and to replicate its (+) RNA into a (−) sense RNA that will be used to encode all the SARS-CoV-2 structural proteins, which are the spike (S), nucleocapsid (N), membrane (M), and envelope (E). After all these processes, the S, N, M, and E structural proteins combine with the viral genome, the virus is assembled into the Golgi apparatus of the host cells, and the mature SARS-CoV-2 virions are released to infect other susceptible cells. ↑, increase; Pp1a/b, polyproteins a/b.

Figure 1 shows the main steps involved in SARS-CoV-2 replication within human cells, from its attachment to its assembly and release.

#### 3.1.1. Inflammation, Immune Dysregulation, and Disease Progression

Antigen presentation is required and an immunological process is necessary to stimulate adaptive immunity. SARS-CoV-2 can induce a hyperinflammatory syndrome in the host, generating a high reactive activity called a cytokine storm, which is mediated by an immune response of the organism. Humoral and cellular immunity is essential for an anti-viral response in coronavirus infection. This mechanism occurs through antigen-presenting cells (APCs)—macrophages and dendritic cells—that capture viral antigens, process and present the antigens to T lymphocytes through the major histocompatibility complex (MHC, or HLA in human)–T cell receptor (TCR) interaction. In addition, MHC-class II is recognized for CD4+ T cells or helper T cells via professional antigen-presenting cells, including dendritic cells, macrophages, some epithelial cells, and B cells. CD8+ T cells recognize non-self-antigens presented by MHC class I on virus-infected cells, which are nucleated. Activation of CD4+ T cells will be necessary to eliminate the virus, as it leads to the maturation of B cells and the generation of neutralizing antibodies [31,32,33].

The severity and mortality rates of COVID-19 are linked and associated with the hyperinflammation the infection induces. This hyperinflammation is a prognostic factor for affected individuals, where inflammation and the innate immune response against the virus are recognized as triggering factors for the disease’s most severe conditions. A meta-analysis by Zeng et al. [34] elucidated the association between inflammatory markers and the severity of COVID-19. The result revealed that the measurement of inflammatory markers is closely linked to the severity of the disease and may correspond to an exemplary methodology for physicians to monitor and assess the severity and prognosis of COVID-19 conditions. In this study, some inflammatory markers were positively related to the severity of the disease, such as C-reactive protein (CRP) at higher concentrations in more severe cases and interleukin-6 (IL-6), which presented at higher concentrations in circumstances that led to death concerning cases of individuals who survived and those with a higher erythrocyte sedimentation rate (ESR). The pathogenesis of severe inflammation that occurs in COVID-19 begins when an inadequate and dysregulated immune response takes over the fight against the viral infection, and the cytokine storm begins, which predominantly and initially affects the lungs. Although IL-6 may be the most predominantly feared inflammatory marker, given that IL-6 concentrations are relatively higher in individuals who died from SARS-CoV-2 compared with those who survived. Individuals affected by COVID-19 who need treatment in intensive care units usually have high levels of interleukin (IL)-2, IL-7, IL-10, tumor necrosis factor α (TNF-α), and other pro-inflammatory and also anti-inflammatory cytokines. The immune system dysregulation leads to patterns of high inflammation, which exacerbate the severity and risk of mortality in patients with COVID-19. This immune dysregulation is attributed to various immune cells. Inflammatory markers and pro-inflammatory cytokines, such as CRP, ferritin, IL-1β, IL-8, TNF-α, and MCP1, show a significant increase in their serum concentrations, and the elevated neutrophil-to-lymphocyte ratio is associated with more severe cases of COVID-19 that can lead to death more easily [31,34,35,36,37].

In addition, the infection promotes a highly inflammatory programmed cell death response called “pyroptosis”, which affects macrophages and other cells of the human immune system. This phenomenon consists of leukopenia processes, with a reduction in the ability of the patient’s immune system to respond to the infection efficiently and correctly. Specific inflammasome pathways that lead to rupture of the plasma membrane of immune cells affected by “pyroptosis” are directly activated with the activation of the inflammasome nucleotide-binding domain leucine-rich repeat NLR and pyrin domain-containing receptor (NLRP3). Therefore, the death of immune cells not only deregulates the host’s immune system in the presence of the SARS-CoV-2 virus but also promotes an intense inflammatory process with the release of intracellular contents into the tissue interstice and with the resulting production of pro-inflammatory cytokines, returning to the vicious cycle of the cytokine storm [31,34,35,36,37,38].

It was observed that in COVID-19, there is a reduction in IFN-I and IFN-III, which allows prolonged viral replication and facilitates OS occurrence. When the redox balance is disrupted, as in the case of COVID-19, free radicals start to be harmful, acting on different cells and damaging DNA. This search for favorable conditions for viral replication leads to excess reactive oxygen species (ROS) and a glutathione deficiency, which, under normal conditions, would reduce ROS and fight against OS. Individuals with chronic low-grade inflammation are known to have a dysregulated innate immune system. Therefore, all these aspects are directly linked with immune dysregulation, as two main features occur in critically ill patients, pro-inflammatory cytokines (especially IL-6) overproduction by monocytes and dysregulation of lymphocytes characterized by CD4 lymphopenia and, subsequently, B-cell lymphopenia in addition to activating the inflammatory response and promoting OS [9,39,40,41].

The progression of the disease was understood through pathological examinations of lungs affected by COVID-19. As the disease progressed, there was an increase in the number of available autopsies, and we can cite some examples in the tracheal mucosa, as in Barnes et al. [42]. In addition, an association between neutrophilic extracellular traps and the presence of lesions in both the lung parenchyma and the airways was reported. Magro et al. [43] built the hypothesis of the role of the complement system as a participant in the pathogenesis of thrombogenic vasculopathy. Barton et al. [44] reported acute lung injuries and microthrombi described in the first report of complete autopsies in Oklahoma (USA).

Gross autopsy findings include increased lung weight, consolidation, vascular engorgement, edema, pleurisy, and mildly erythematous trachea. At the microscopic level, diffuse alveolar damage, lymphocyte infiltration in interstitial regions, giant pneumocytes adjacent to multinucleated giant cells, pneumocyte hyperplasia and desquamation, which leads to an apparent viral cytopathic effect, intra-alveolar fibrin deposition, exudate formation, lymphocytic inflammation, loose connective tissue within the alveolar ducts and bronchioles, alveolar fibrin surrounded by fibroblasts, bronchial epithelial denudation, loss of cilia, squamous metaplasia, and hyaline membrane formation were the most commonly observed histopathological changes [31,45]. Studying COVID-19 autopsy cases, Martínez-Colón et al. [46] also discovered that SARS-CoV-2 RNA was present in adipocytes and was associated with an inflammatory infiltration. These authors’ biochemical analyses also revealed that a particular group of macrophages residing in adipose tissue was also infected by SARS-CoV-2 in AT. Although mature adipocytes were found to be susceptible to SARS-CoV-2 infection, the macrophages showed only an incomplete infection. Nevertheless, the virus was able to activate inflammatory responses within both the infected macrophages and adjacent preadipocytes.

In general, there is a tendency for the formation of microthrombi, typically located in small- and medium-sized pulmonary arterial vessels, contributing to the organ dysfunction and mortality of COVID-19. In vitro studies also demonstrated that SARS-CoV-2 can directly infect a modified human organic blood vessel via the ACE2 receptor. This suggests a direct viral invasion by SARS-CoV-2 that triggers endothelium inflammation contributing to endothelial injury in COVID-19. In most cases, there is also a mononuclear inflammatory infiltrate in the lung parenchyma, composed mainly of lymphocytes and macrophages. CD4+ T cells aggregate around small vessels that often contain microthrombi. Many CD68+ macrophages and multinucleated cells were also identified and located in the alveolar lumen, and the presence of macrophages is associated with the evolution and worsening of the disease. This leads back to the hyperinflammatory syndrome summarized in SARS-CoV-2-induced hemophagocytic lymphocytosis, including macrophage activation with a storm of cytokines and a massive presence of natural killer cells and CD8+ cells [31,34,35].

#### 3.1.2. COVID-19 and Comorbidities

It is important to note that people with comorbidities such as diabetes, obesity, and uncontrolled blood pressure are at higher risk for severe COVID-19 and should take extra precautions to prevent infection [47]. In particular, people with diabetes have impaired immune function, which can increase susceptibility to infection [48,49,50]. Obesity can also compromise immune function and ventilation, making individuals more vulnerable to COVID-19 [51,52,53,54]. Uncontrolled hypertension is associated with increased expression of ACE2 receptors, which can increase susceptibility to infection [55,56,57,58,59].

Cardiovascular diseases, liver injury, and malignancy also play important roles in COVID-19 severity [47]. Patients with pre-existing cardiovascular disorders possess a higher risk of developing severe COVID-19 symptoms and complications. The presence of ACE-2 receptors on cardiac muscle cells suggests the involvement of the cardiovascular system in SARS-CoV-2 infection [60,61,62,63,64]. COVID-19 patients may also exhibit abnormal liver enzyme secretion [65,66,67]. Patients with malignancy are also at a higher risk of developing COVID-19 due to a weakened immune response [68,69].

SARS-CoV-2 can also affect the kidneys, leading to acute kidney injury (AKI) in a small percentage of cases and raised blood urea nitrogen and proteinuria levels. Patients with pre-existing renal diseases are more susceptible to COVID-19 infection due to increased ACE2 expression [70,71,72,73]. Asthma, conversely, does not seem to be directly associated with COVID-19, but asthmatic smokers, particularly geriatric ones, are at a higher risk of developing the serious disease [74,75,76,77]. People with other pulmonary chronic disorders, such as chronic pulmonary obstructive disease (CPOD), have an increased risk of the occurrence of severe COVID-19 symptoms due to elevated expression of ACE2 receptors in the lungs, weak immunity, and continual mucus production, leading to blockage of air passages [78,79,80,81,82].

### 3.2. Adipokines in COVID-19

Adipokines such as apelin, omentin, and adiponectin are primarily organokines derived from adipocytes. These are tissue-induced mediators and regulated systemic inflammatory factors with metabolic effects throughout the human body. Adipokines can influence obesity progression and its associated comorbidities. Although many adipokines provide anti-inflammatory and cardioprotective actions, these can also be involved in inflammatory diseases such as atherosclerosis and COVID-19. In rheumatoid arthritis, adipokines influence immune cells, synovial tissue cells, cartilage, and bones. Among dermatological conditions, adipokines can influence melanogenesis, hair growth, and wound healing and alter psoriasis, atopic dermatitis, and melanoma pathogenesis [83,84,85].

#### 3.2.1. Adiponectin

The most abundant secreted adipokine in humans is adiponectin, encountered in healthy individuals’ plasma at 4 to 37 µg/mL (corresponding to approximately 0.01% to 0.05% of total plasma proteins). Adipocytes from healthy AT are the primary sources of this molecule, but other cells such as myocytes, cardiomyocytes, hepatocytes, endothelial cells, osteoblasts, and pituicytes can also be producers of adiponectin. Adiponectin principally binds to AdipoR1- and AdipoR2-specific receptors, stimulating the peroxisome proliferator-activated receptor α (PPAR-α) and adenosine monophosphate (AMP)-activated protein kinase (AMPK) signaling pathways. Generally, adiponectin comprises many metabolic and non-metabolic effects: elevated fatty acid oxidation and glucose uptake, reduced apoptosis, increased vasodilation, and reduced inflammation and fibrosis [86,87].

In COVID-19 patients, adiponectin levels were found to be mostly lower compared with controls, especially in respiratory failure, which may reflect the associations of adiponectin with the severity of COVID-19. However, among mild, moderate, and severe COVID-19 patients, adiponectin levels were higher in severely diseased individuals compared with mild and moderately affected individuals. In correlations with other adipokines, higher adiponectin serum levels were demonstrated to reflect the inflammatory burden during the disease. The adiponectin/leptin ratio also significantly discriminates against COVID-19-related pneumonia, even in non-obese individuals (with BMI (body mass index) values ˂ 25 kg/m^2^). Other studies evidenced that SARS-CoV-2 can replicate into the adipocytes and cause AT inflammation, which alters mRNA expression in these cells and leads to AT dysfunction with modified adipokines secretion. Lower levels of adiponectin were found to be correlated with the occurrence of severe hyperglycemic events during COVID-19. Additionally, obese individuals were more susceptible to serious COVID-19 outcomes than those with average weight. Serum adiponectin levels significantly and positively correlated with IL-6, ceramides, and glycerophospholipids serum levels during COVID-19 [17,88,89,90,91,92].

Across the COVID-19 pandemic, much has been said about the “adiponectin paradox” and how this condition can be caused during SARS-CoV-2 infection, principally among infected obese people. It is a fact that higher serum adiponectin levels drive insulin resistance (IR) down [93]. It is also known that during COVID-19, hyperglycemia happens principally among severely ill patients [94,95]. As commented above, the serum levels of adipokine adiponectin among COVID-19 patients differ primarily according to the patients’ disease severities. Among diseased individuals with hyperadiponectinemia and direct AT infection, there is speculation that the consequent hyperglycemia directly correlates with the occurrence of a SARS-CoV-2-inflammation-derived adiponectin paradox. Functionally, the adiponectin requires at least one of the AdipoR1 or AdipoR2 receptors to make its signal transduced, such as the glycosylphosphatidylinositol-anchored molecule, T-cadherin. In obesity, there is a reduction in adiponectin synthesis due to AT dysfunction but also an increase in the production of the glycosyl phosphatidylinositol-phospholipase D (GPI-PLD) enzyme. This enzyme hydrolyzes T-cadherin, consequently reducing the functional, active T-cadherin molecules, and promotes a decrease in adiponectin sequestration by the responsive tissues. This reduced sequestration causes an even more substantial reduction in adiponectin’s signal transduction and increases adiponectin’s presence in the bloodstream. This process, therefore, creates the paradox that although adiponectin serum levels are high, insulin sensitivity is remarkably impaired due to the strongly decreased adiponectin signal transduction. Among infected individuals with hyperadiponectinemia, it is believed that the adiponectin paradox can happen due to the hyperproduction of pro-inflammatory cytokines, which also leads to the cytokine storm [96,97,98].

The hyperglycemia observed in patients with COVID-19 was associated with many phenomena that worsen the disease’s prognosis, such as the need for mechanical ventilation and admission to the intensive care unit (ICU). One of those phenomena is the loss of the body’s redox homeostasis, which is associated with increased production of ROS; this phenomenon potentializes OS and contributes to the increase in nitrosative stress (NSS). In a comparative cohort and analytical study, 61 COVID-19 patients with and without comorbidities and 25 healthy individuals were evaluated for glycemia, lipid peroxidation (LPO), nitrites assays, and total antioxidant capacity (TAC). All glucose, LPO, and 3-nitrotyrosine assessments were elevated in the infected subjects. In turn, the TAC was decreased in the same patients. These alterations were highly associated with fatal outcomes, principally in individuals pre-affected by cardiometabolic abnormalities, such as metabolic syndrome (MetS) [9,94,99,100].

**Figure 2:**
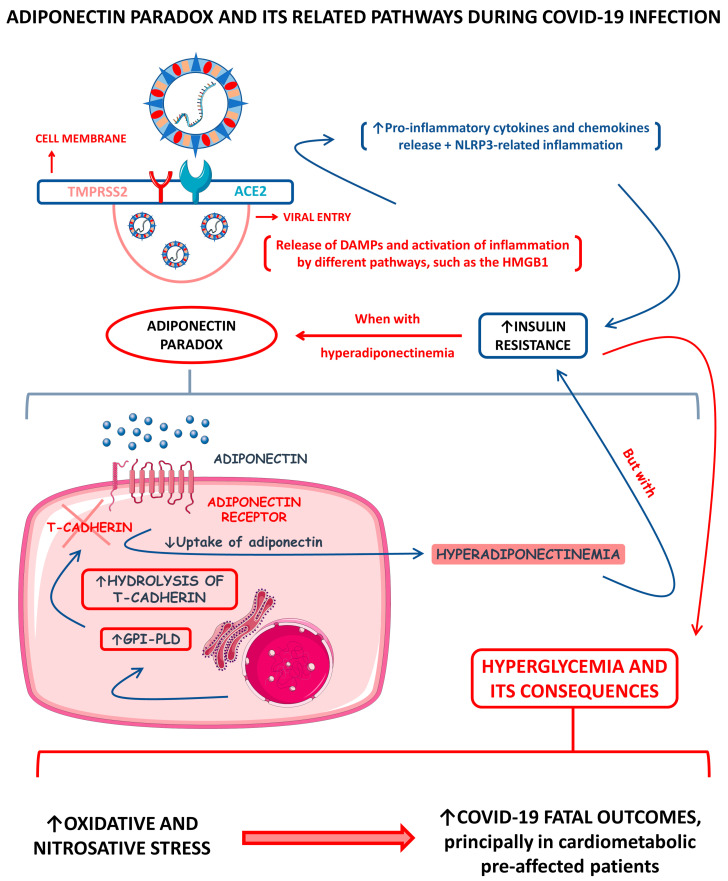
Adiponectin paradox and its related pathways during COVID-19 infection. ↑, increase; ↓, decrease; ACE2, angiotensin-converting enzyme 2; DAMPs, damage-associated molecular patterns; GPI-PLD, glycosylphosphatidylinositol-phospholipase D; HMGB1, high-mobility group B 1; NLRP3, NLR (nod-like receptor) family pyrin domain containing 3; and TMPRSS2, type II transmembrane serine protease 2.

Figure 2 shows a schematic representation of the adiponectin paradox and its related pathways during COVID-19.

#### 3.2.2. Apelin

Apelin is produced mainly by the AT and exerts endocrinological roles among different organs and biological systems. The apelin system comprises the apelin receptors and two endogenous ligands: the apelin and the elabela/toddler molecule. In the nervous system, apelin contributes to the inhibition of vasopressin release, as well as regulates water intake. It improves vasodilation and decreases blood pressure in the blood vessels, promoting angiogenesis and anti-thrombotic effects. In the skeletal muscles, apelin augments glucose uptake and increases the body’s insulin sensitivity. In the kidneys, apelin promotes augmentation of the renal blood flow and augments diuresis, and during chronic kidney diseases, it diminishes inflammation and contributes to decreased fibrosis formation. In the AT, apelin promotes AT browning and increases mitochondrial biogenesis. In the heart, apelin improves inotropism, decreases the preload and afterload charges, reduces cardiac hypertrophy and fibrosis during aggression, and promotes anti-arrhythmic effects [101,102,103].

There is still a lack of complete clinical studies on the roles of apelin during COVID-19. However, much has been discussed on how potential therapies involving apelin could be associated with better COVID-19 prognosis and cure expectancy. These discussions lined up with the utilization of the ACE2 receptor of the host cells by the SARS-CoV-2 in its entry. When the SARS-CoV-2 binds to the ACE2 receptor, the expression of this receptor is downregulated; consequently, the Ang-II molecules concentration increases due to the occupation of the ACE2 by the virus. Clinical evidence suggests that when Ang-II concentration is elevated, this molecule starts to mediate acute lung and cardiovascular injuries and cause prothrombotic events during COVID-19. Since apelin and its analogs suppress the Ang-II production and downregulate the ACE2 upregulation, apelin as a potential therapeutic could be associated positively with the treatment against SARS-CoV-2 [104,105].

Some studies also postulated that the apelin singling pathways could be associated with cardiorenal implications during COVID-19. According to the study of Li et al. [16], abnormal apelin-ACE2 and SGLT2 (sodium-glucose cotransporter 2) signaling contributes to adverse cardiorenal injuries in patients with COVID-19. These authors described their theory in an RNA-sequencing and human-based study that comprised cardiomyocytes from people infected with SARS-CoV-2. The results showed decreased ACE2 and apelin expression levels in the infected cardiomyocyte and increases in the SGLT2 and endothelin-1 levels. Following the same results, humans infected by SARS-CoV-2 also had remarkably lower ACE2 and apelin levels and higher SGLT2 and endothelin-1. Intriguingly, these alterations were linked to downregulated IL-10, Superoxide dismutase 2 (SOD2), catalase levels, and upregulated profibrotic and pro-inflammatory gene expression, which upregulated cardiorenal fibrosis stimuli due to inflammatory mechanisms in the studied samples. Despite other conclusions, the downregulation of apelin and ACE2 contributed cardinally to the upregulation of SGLT2, endothelin-1, and pro-inflammatory cytokines. These results elevate apelin to a potential therapeutic target against COVID-19-mediated cardiorenal injury among infected patients, such as cardiorenal fibrosis.

#### 3.2.3. Leptin

Leptin is an adipokine produced mainly by activating obese-related genes in susceptible adipocytes. This molecule can act as a hormone and a cytokine. Its effects are triggered by binding to the leptin receptor (Lep-R), a class I receptor cytokine family that mediates the phosphorylation and activation of the signal transducer and transcription activator of transcription 3 (STAT3) signaling pathways. Leptin is associated with regulating energy balance, leading to anorexigenic and orexigenic homeostasis. However, the roles of leptin are vast, principally because the Lep-R is present is many types of cells, including immunological ones. Leptin helps trigger the immune response through the Janus kinase-signal transducer and activator of transcription (JAK-STAT), and nuclear factor kappa B (NF-kB)-dependent pathways and leptin-deficient or leptin receptor (Lep-R)-deficient mice are immunocompromised. Due to these effects on the immune system, leptin can stimulate the production of many pro-inflammatory cytokines, such as TNF-α, IL-1, and IL-6, and was found to stimulate the proliferation of circulating monocytes, T helper cells (Th), and natural killer cells (NK). Due to the above information, two crucial questions that summarize the SARS-CoV-2 appearance are how the leptin levels would behave during COVID-19 infection and whether leptin levels could be linked to the occurrence of the cytokine storm during COVID-19, principally because obese individuals present leptin resistance. This phenomenon is closely associated with cytokine secretion dysregulation [106,107,108].

Studies have shown leptin levels could predict disease severity and significantly correlate with decreased lymphocyte count and disease progression in severe COVID-19 individuals. In infected patients, leptin serum levels appeared to be considerably higher than in healthy controls, especially in women. However, these levels did not correlate significantly with the mortality rates. Molecularly, studies showed that during COVID-19, leptin negatively correlated with IL-6 and IL-1β serum levels. Nevertheless, other studies demonstrated that leptin could effectively produce M1 macrophage polarization in COVID-19-infected individuals through upregulation of pro-inflammatory cytokines production and of surface monocyte activation markers’ mechanistic actions principally by the stimulation of the STAT-3 and NF-kB signaling pathways [109,110,111].

Results from the autopsy of lungs from obese COVID-19 patients showed upregulation of the lymphocyte-specific kinase (LCK) and early growth response 2 (EGR2) genes. During symptomatic lung diseases, leptin is the main stimulator of LCK and EGR2 genes, which exacerbates inflammation and activates monocytes, respectively. It is known that the continuous activation of these genes can cause abnormal ventilation, immune dysregulation, and excessive lung tissue remodeling [112,113].

People with extensive visceral adipose tissue (VAT) also present IR, hypertension, and leptin resistance. These conditions bring obese people to a constant low-grade pro-inflammatory state, which is accelerated when the obese get infected by SARS-CoV-2. The ACE2 receptor also suppresses leptin levels by the MrgD-receptor/c/Src/p38MAPK pathway, which means that with SARS-CoV-2 compromising the function of the ACE2 receptor, the leptin levels can rise. These events probably result in a hyperinflammatory state locally in the pulmonary tissue infected by SARS-CoV-2, which involves local leptin receptors and the proper ACE2 disbalance. This hyperinflammatory state caused partly by leptin can be explained by the clinical studies that encountered higher leptin serum levels predicting disease severity during COVID-19 infection [114,115,116].

One of those required explanations can be that leptin resistance impairs IFN-I and IFN-III early responses during SARS-CoV-2 infection. The condition of chronic hyperleptinemia and elevated serum levels of pro-inflammatory cytokines presented by obese people induce the suppressor of cytokine signaling 1 (SOCS1) and suppressor of cytokine signaling 3 (SOCS3) pathways, which properly deactivate the leptin receptors among immunological cells, as well as the SOCS1/3 sensitive cytokine receptors in the same cells. These actions impair IFN-I and IFN-III early responses against SARS-CoV-2. However, during the COVID-19 infection, an upregulation of the SOCS1/3 signaling happens, and the presence of SARS-CoV-2 significantly boosts these pathways’ actions. The results summarize a delayed but over-reactive immunological response against the virus, characterized by high-grade inflammation, hypercoagulation, and endothelial damage. This triad would thus lead to COVID-19 higher severity [117,118].

**Figure 3:**
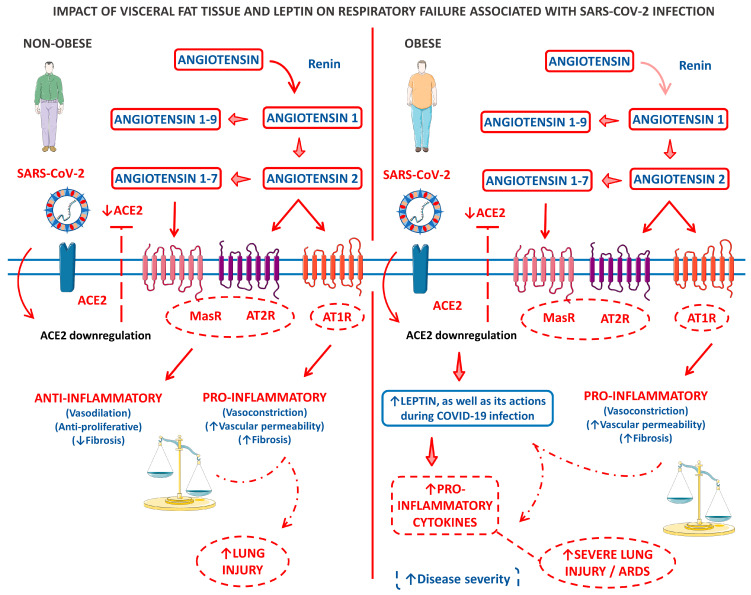
A panel that outlines a clinical and biological framework regarding the impact of visceral fat tissue and leptin on respiratory failure associated with SARS-CoV-2 infection. The left panel describes a scenario where an infected individual who is not obese experiences limited lung injury due to a disruption in the balance of ACE2-ATII. In contrast, the right panel illustrates the pro-inflammatory baseline state of patients with central adiposity, particularly those with metabolic syndrome. This baseline state is further intensified by an imbalance in ACE2-ATII and an increase in leptin production induced by ACE2 deficiency. ↑, increase; ↓, decrease; ACE2, angiotensin-converting enzyme 2; AT1R, angiotensin 1 receptor; AT2R, angiotensin 2 receptor; and MasR, Mas receptor.

Figure 3 shows a model of the clinical and biological framework on the role of leptin derived from the VAT of obese patients in COVID-19-related respiratory failure.

#### 3.2.4. Progranulin

Progranulin (PGRN), also known as a granulin–epithelin precursor (GEP), pro-epithelin, acroglanin, or GP88, is a cysteine-rich secreted hormone expressed by immune and endothelial cells, neurons, and adipocytes that act as an organokine principally by exerting anti-inflammatory effects. However, PGRN is an adipokine that exerts neuroprotection and has roles in cardiovascular diseases. PGRN hyperexpression is highly associated with obesity, and hyperprogranulinemia is involved in developing various metabolic disorders, such as IR [119,120,121]. Against liver fibrosis, PGRN acts by downregulating the inflammatory response in the hepatic tissue, which diminishes the fibrotic stimuli [122]. Against dementia, PGRN induces neuroprotection due to its effects against neuroinflammation [123,124].

It is known that in COVID-19, the serum levels of PGRN were significantly higher compared with healthy controls. Besides many other biochemical parameters, such as CRP, PGRN was a better biomarker for the COVID-19 prognosis [125]. PGRN was also found to have relationships with the circulating vascular cell adhesion molecule-1 (sVCAM) in a cohort of COVID-19 patients investigated by Yao et al. [126].

### 3.3. Myokines in COVID-19

During the last few years, scientists all around the globe have discovered that the skeletal muscles are not only for locomotion or responsible for the body’s posture but also for the production of unique molecules that control vital functions of the human body. Myokines can exert autocrine, paracrine, or endocrine effects and mediate communication between skeletal muscles and other tissues and organs such as the adipose tissue, brain, nervous tissue, bones, liver, pancreas, gut, skin, and even pancreas. Besides personal effects, myokines can also exert crosstalk and mediate actions with other organokines on the body’s homeostasis. Metabolically, myokines can control lipid, glucose, and bone metabolisms, in addition to the white adipose tissue (WAT) browning. Neurologically, myokines can influence cognition and the skeletal muscles; myokines help principally in hypertrophy [127,128].

#### 3.3.1. Irisin

Irisin is a myokine induced by exercise that has been associated with energy expenditure promotion by stimulating the browning of the WAT principally through upregulation of the uncoupling protein 1 (UCP1). It is secreted mainly by myocytes in response to exercise and exerts beneficial effects on the skeletal muscles. Irisin is widely distributed in the heart, cerebrospinal fluid, and paraventricular nucleus of the hypothalamus. Neurologically, irisin modulates synaptic plasticity and increases memory in Alzheimer’s disease animal models. In the bones, irisin promotes osteocyte survival and augments sclerostin secretion. Irisin also plays a role in IR by principally improving the phosphoinositide-3-kinase/protein kinase b (PI3K/AKT) insulin signaling pathway and in inflammation by suppressing the phosphorylation of the mitogen-activated protein kinase (MAPK) and lowering the NF-kB activation [129,130].

Studies have found that irisin can modulate genes associated with severe COVID-19 infection in human subcutaneous adipocytes. In COVID-19 patients, irisin serum concentrations were significantly decreased in patients with homeostasis model assessment for insulin resistance (HOMA-IR) values ≤ 3 compared with healthy controls. These reduced levels may be derived from the SARS-CoV-2 potential involvement of the ACE2/Ang-(1–7)/Mas in downregulating the production of PGC1α, therefore downregulating irisin expression and liberation from the skeletal muscle cells. It was found that irisin treatment significantly increased by up to three-fold the expression levels of TRIB3 transcription among cultured human adipocytes and decreased the levels of other stimulatory genes. Therefore, the viral entry in the human cells would be compromised. Researchers also hypothesize that the roles of irisin during COVID-19 go beyond genetic modulation, principally among pre-infection exercise-active patients. Irisin can positively modulate AMPK during predominantly aerobic exercise. AMPK induces the endothelial nitric oxide synthase (eNOS), which increases nitric oxide (NO) bioavailability and inhibits the palmitoylation of the SARS-CoV-2 S protein, leading to specific and immediate protection against SARS-CoV-2. Irisin also protects mitochondrial functions in endothelial cells principally by antagonizing the renin–angiotensin pro-inflammatory signaling pathways, which can probably cover the infected individuals against the SARS-CoV-2-mediated fatal cardiovascular outcomes [21,131,132,133,134].

#### 3.3.2. Myostatin

Myostatin is well known as the only myokine with reduced levels following exercise training. This molecule causes muscle protein degradation and inhibits muscles’ satellite cells’ function, which is positively correlated with sarcopenia occurrence. Myostatin belongs to the transforming growth factor beta superfamily (TGF-β), and even during embryogenetic development, this myokine induces limitations to muscle growth. Its receptor is the activin type 2 receptor (ACTRIIB), and the catabolic cascades activated by myostatin are the SMAD1/SMAD2 and forkhead box transcription factors family (FOXO) 1, 2, and 3. Anabolic cascades, such as the mTOR and AMPK, are inactivated by myostatin. Besides showing a central role in regulating muscle growth and atrophy, myostatin also regulates metabolic factors and is positively associated with obesity and IR [135,136]. In COVID-19 patients, myostatin is a myokine related to glucose and lipid metabolic disturbances and acute sarcopenia onset among the infected elderly [18,137].

Besides the presence of IR, lipid dysregulations, especially of the (±)5-HETE, propionic acid, (±)12-HETE, and isobutyric acid, were also correlated with metabolic disturbances that could lead to hyperglycemia in COVID-19 patients. This myokine is also profoundly and mechanistically affected by the RE1-silencing transcription (REST) factor during the SARS-CoV-2 infection. The REST factor has also increased expression during COVID-19. Many authors have suggested that the REST factor overexpression promoted by myostatin acts against metabolic glycemic control in COVID-19 patients. Myostatin can also act with the REST factor to overexpress the lipids mentioned above, which could also contribute to hyperglycemia. Based on the fact that myostatin actions in the human body can be altered by other endocrine molecules such as the REST factor and that these alterations can influence metabolic regulatory measures such as the glycemic control of the diseased patients, myostatin can be a potential therapeutic target for the prevention of COVID-19-induced metabolic complications [137,138].

In muscles, myostatin is correlated with developing new-onset acute sarcopenia in elderly COVID-19 patients. The SARS-CoV-2 infection not only leads to high-grade severe inflammation but also to a highly catabolic state in which significant metabolic changes influence homeostasis, significantly downgrading the structure, amount, and function of myocytes. Due to these events, new-onset acute sarcopenia has become a muscular condition that primarily impacts the in-hospital prognosis of interned patients and increases the vulnerability of principally elderly patients to post-COVID-19 function and physical impairments. The molecular mechanisms involved in this new onset acute sarcopenia are the atrogin-1/muscle atrophy F-Box (MaFbx)/muscle ring finger 1 (MuRF1) pathway, the insulin-like growth factor 1–protein kinase b–mammalian target of rapamycin (IGF-1-AKT-mTOR) and, principally, the myostatin pathway. The MaFbx/MuRF1 is a well-established inducer of sarcopenia. The IGF-1-AKT-mTOR induces muscle hypertrophy when stimulated, but when inhibited during disease immobilization, it causes muscle degeneration [18,139,140].

In COVID-19 patients, myostatin-related new-onset sarcopenia happens due to the myostatin signaling that activates the phosphorylation of the SMAD proteins. The activated SMAD proteins form complexes that are co-mediators of the bone morphogenic proteins (BMPs) signaling pathways. When myostatin activation is elevated, such as during COVID-19 infection, the SMAD complexes become less unavailable to the BMPs, and the skeletal muscles lose proteins. Taken together, all events during new onset acute sarcopenia in COVID-19 infection induced muscular autophagy via increased mitochondrial dysfunction and decreased mitochondrial biogenesis, which decreases skeletal muscles synthesis and increases not only myofibrillar breakdown but also skeletal muscles degradation [18,140,141,142].

#### 3.3.3. Brain-Derived Neurotrophic Factor

Brain-derived neurotrophic factor (BDNF) is a small dimer protein that belongs to the neurotropic protein family of the central nervous system. This molecule is highly expressed in the brain, muscles, and endocrine system, although recent research evaluated its expression in the bones and cartilage. Besides other roles, BNDF plays its most important actions in the survival, proliferation, and differentiation of neurons and glial cells [143,144,145].

BDNF can provoke mitochondrial fission and clearance in the skeletal muscles via two different pathways, the Protein Kinase AMP-Activated Catalytic Subunit Alpha 2/adenosine monophosphate (AMP)-activated protein kinase-PTEN-induced kinase 1-PRKN/Parkin (PRKAA/AMPK-PINK1-PRKN/Parkin) and Protein Kinase AMP-Activated Catalytic Subunit Alpha 2-Dynamin-1-like protein/dynamin-related protein 1-Mitochondrial fission factor (PRKAA-DNM1L/DRP1-MFF). As a myokine, muscle-generated BDNF is implicated in maintaining muscle’s mitochondrial quality, and its expression during obesity might involve metabolism impairments. Muscle-specific BDNF knockout mice displayed defective mitophagy and mitofission, impaired lipid handling, and mitochondrial elongation [146,147,148].

In COVID-19 patients, BDNF serum levels were found to be significantly lower when in comparison with healthy controls. Furthermore, serum concentrations of BDNF during COVID-19 infection were found to adhere to age-related characteristics, with higher levels observed in younger individuals compared with the elderly. When analyzed with other organokines, the BDNF/adiponectin ratio predicted a significant risk for poor prognosis of COVID-19 in a cohort of 145 hospitalized COVID-19 patients (78 non-obese and 67 obese). However, among COVID-19 patients, BDNF serum levels were not statistically significant between patients with and without neurological manifestations derived from the infection [149,150,151]. Moreover, Asgarzadeh et al. [150] conducted a study and reported a significant negative correlation between serum levels of BDNF and the need for oxygen therapy among their sample dataset of COVID-19 patients. This finding strongly suggests a crucial link between regulated BDNF and hypoxia in promoting COVID-19 symptoms such as fever and dyspnea. In cases where patients manifested central nervous system symptoms, BDNF levels were lower than those with fever and dyspnea.

The altered hemodynamics during COVID-19 has also been implicated to be derived from the ACE2 dysregulations. Although without complete clarity, some authors hypothesized that ACE2 impairments were also responsible for the dysregulations of some essential proteins, such as Mas proteins, that regulate normal brain function due to the stimulation for the production of neurotrophic factors, such as the BDNF. This molecule exerts critical roles in neurodevelopment and neurogenesis, as well as in inhibiting neurodegeneration and maintaining normal behavior by modulating mood stability and cognition function. According to this hypothesis, decreases in the ACE2 number and activity during COVID-19 could trigger neural disturbances that lead to acute and long-term neural alterations and sequels, respectively. The theory proposes that decreased ACE2 activity results in reduced production of BDNF. The downregulation of BDNF can lead to increased oxidative stress, neuroinflammation, and neuro-apoptosis, which can cause long-lasting mental disorders such as anxiety, depression, and cognitive impairment. These effects could persist throughout the illness or as a sequel [152,153].

**Figure 4:**
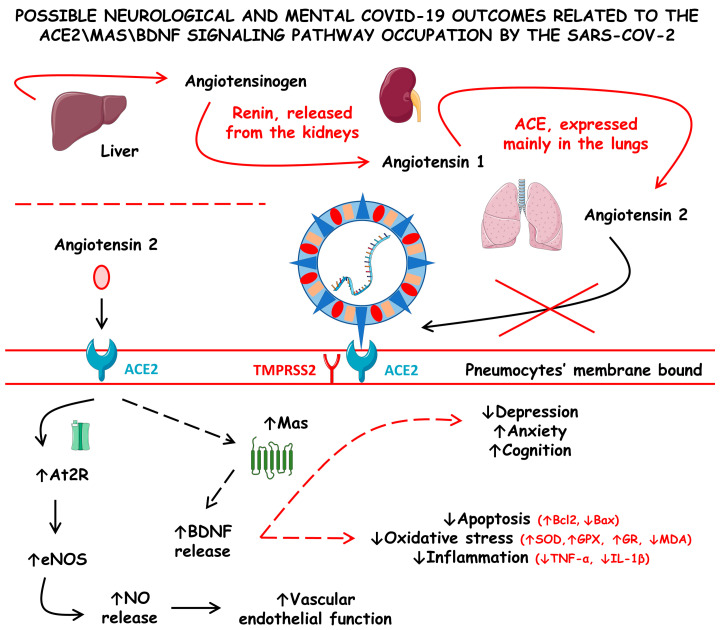
Possible neurological and mental COVID-19 outcomes related to the angiotensin-converting enzyme 2/Mas/brain-derived neurotrophic factor (ACE2\Mas\BDNF) signaling pathway occupation by SARS-CoV-2. ACE, angiotensin-converting enzyme; At2R, angiotensin 2 receptor; Bax, Bcl-2-associated X protein; Bcl2, B-cell lymphoma 2; eNOS, endothelial nitric oxide synthase; GPX, glutathione peroxidase; GR, glutathione reductase; IL-1β, interleukin 1 beta; MDA, malonaldehyde; NO, nitric oxide; SOD, superoxide dismutase; TMPRSS2, type II transmembrane serine protease 2; and TNF-α, tumor necrosis factor alfa; ↓ increase; ↑ decrease.

Figure 4 shows the possible neurological and mental COVID-19 outcomes related to the ACE2\Mas\BDNF signaling pathway occupation by SARS-CoV-2.

### 3.4. Osteokines in COVID-19

Osteokines are secreted by bone cells such as osteoblasts, osteocytes, and osteoclasts that exert effects locally in bones and systemically in other tissues and organs. Osteokines exert endocrine, autocrine, and paracrine actions. In skeletal muscles, they mainly have positive effects such as promoting post-contraction glucose uptake by myocytes through insulin-dependent mechanisms, increasing the mitochondrial bio-genesis of muscles, facilitating the translocation of glucose transporters-4 (GLUT-4) from the cytoplasm to the membrane, and regulating the oxidation of free fatty acids. Additionally, they slow down the aging process [154,155,156,157].

#### Osteopontin

Osteopontin (OPN) belongs to an N-link integrin-binding ligand glycoprotein family and is present in low concentrations as a soluble cytokine in healthy individuals [158,159,160]. Although it was first identified in the bone, it is found on multiple cell surfaces and may play a role in bone remodeling and immune modulation [161]. The pathophysiological mechanism of OPN overexpression in pathologies such as atherosclerosis, diabetes, and tumor progression is not yet fully understood [162].

OPN has three integrin binding sites, the RGD-, SVVYGLR-, and ELVTDFTDLPAT- binding domains, in addition to two Ca2+ binding sites (CaBS) [163]. Recently, a new receptor for OPN different from those currently known (integrins and CD44) was identified: the ICOS-L receptor expressed by antigen-presenting cells [164]. Modulation of OPN production occurs through different signaling pathways, such as protein kinase C (PKC) that can suppress OPN release in Src-/- fibroblasts, which is stimulated by the epidermal growth factor [160].

Regarding immune modulation, OPN can act as a chemotactic factor in the migration of neutrophils, mast cells, and macrophages, in addition to increasing the inflammatory response of TH1 cells by suppressing IL-10 production in Th2 cells [165,166]. Macrophages, eosinophils, NK, neutrophils, dendritic cells (DC), and T and B lymphocytes can express OPN, and OPN can stimulate the production of immunoglobulins by B cells [167,168]. Elevated levels are found in patients with systemic inflammation and non-COVID-19-related ARDS and are related to increased mortality in these patients. On the other hand, studies have shown a strong relationship between high levels of OPN and the prognosis of patients with COVID-19 treated in the ICU [169]. In inflammatory lung diseases that progress to pulmonary fibrosis, such as COVID-19, there is increased expression of OPN, as this organokine modulates fibrogenesis and collagen remodeling. Therefore, dysregulation of OPN levels may contribute to the immunopathogenesis of COVID-19 and disease severity with the development of a fibrotic phenotype [170].

In an experimental study with mouse models, authors analyzed the effects of the lipopolysaccharide (LPS) and spike glycoprotein on OPN secretion by macrophage/monocyte lineage cells. It was demonstrated that the suppression of OPN secretion by LPS is dose dependent, a characteristic triggered by the cytokine storm. Furthermore, OPN upregulated the cytokines of the Th1 response, and its co-injection with RB spike glycoprotein partially reversed the spike glycoprotein-dependent chemokine induction. These data suggest the hypothesis that a cytokine storm is manifested when the agent successfully suppresses OPN expression. Furthermore, macrophages can produce levels of OPN capable of inducing an efficient Th1 response. However, a high enough viral load, necessary for initiating a cytokine storm, can suppress most macrophages and reduce OPN levels [171]. OPN acts to increase IL-12 secretion and decrease IL-10, acting as an inducer of Th1 responses. This increased IL-12 secretion modulates the generation of TCD8+ memory cells. Despite this, OPN expression is generally absent in massive cytokine release syndromes, providing a favorable environment for expanding the SARS-CoV-2 virus in COVID-19 [172].

### 3.5. Hepatokines in COVID-19

The liver and its hepatocytes are responsible for maintaining mainly energy metabolism via regulation in a diversity of different metabolic conditions, including exercise, fasting diet, MetS, obesity, and diabetes under energy storage in the form of glycogen or energy utilization stimuli. In recent years, evidence has suggested that hepatocytes act throughout the human body by producing hepatokines that also have autocrine, endocrine, and paracrine functions. Hepatokines exert more significant interference in the fat and striated skeletal muscle tissues [23,173].

#### 3.5.1. Pentraxin 3

Pentraxin 3 (PTX3) is a long prototypic humoral soluble pattern recognition molecule of the pentraxin family recognized as an hepatokine. This molecule exerts fundamental roles in innate immune response and inflammation, which is pivotal during tissue damage and remodeling. PTX3 is present in the occurrence and development of many auto-immunological diseases, such as rheumatoid arthritis, systemic sclerosis, systemic lupus erythematosus, and multiple sclerosis, as well as during microbial moieties. PTX3 acts as a functional ancestor of antibodies, and data from animal studies indicated that this hepatokine could exert cardioprotective effects, principally against atheroma formation. Unlike the classical short pentraxins CRP and serum amyloid P component (SAP), which are predominantly expressed and released by the hepatocytes, PTX3 can be synthesized by myeloid and other stromal bone marrow cells. PTX3 also has a role in cancer development and progression. Many studies have identified PTX3 promoting increased cancer cells proliferation and migration, as well as decreased cancer cell apoptosis across different types of cancers, such as esophageal squamous cell carcinoma, lymphomas, leukemias, glioblastomas, and renal and prostate cancers [134,174,175,176,177,178,179,180].

In COVID-19 patients, PTX3 has been extensively studied since the beginning of the pandemic, and it was found to be at higher levels during the disease compared with healthy controls. PTX3 also served as a potent independent prognostic predictor of short-term mortality during COVID-19. Associated with age, “Age, PTX3” was found to be the best binary signature related to ICU mortality at 28 days of hospitalization [181,182,183]. Studies have also evaluated whether a specific genotype of PTX3 could be associated with the macrophage activation burden during COVID-19. The AG genotype was most effectively related to the macrophage activation syndrome occurrence. High levels of PTX3 were found to be positively correlated with coagulopathies in COVID-19 patients. This hepatokine was also observed to be positively associated with serum levels of D-dimer in hospitalized patients. PTX3 serum levels at admission were assessed to be helpful as a potent predictor of disease severity [184,185]. It was discovered that PTX3 was associated with the inflammatory status of COVID-19 patients, and treatment with siltuximab reduced the levels of this hepatokine in the blood, resulting in improved ventilation and increased survival chances [186].

Stravalaci et al. [187] conducted a systematic investigation to study the interactions between human humoral fluid-phase pattern recognition molecules (PRMs) and SARS-CoV-2. PTX3 was found to bind the SARS-CoV-2 N protein; therefore, this innate immunity molecule can probably exert resistance to COVID-19 and partially inhibit its pathogenesis [188].

#### 3.5.2. Fetuin-A

Being abundantly secreted by the hepatocytes and adipocytes, fetuin-A is a member of the cystatin protease inhibitor superfamily. Otherwise known as a2-HS-glycoprotein (AHSG), this organokine is involved in many physiological and pathophysiological events in the human body. It can also perform crosstalk by controlling metabolism and affecting calcium and bone regulation. Fetuin-A can induce lipids-derived IR by binding the toll-like receptor 4 (TLR-4) and direct-IR by directly inhibiting the insulin receptor tyrosine kinase. Studies have indicated that fetuin-A is associated with polycystic ovary syndrome and type 2 diabetes mellitus (T2DM) development.

Additionally, high serum concentrations of fetuin-A can correlate with the induction of pro-inflammatory signaling pathways. Together with these findings, fetuin-A and its related genes are essential in developing MetS parameters and the pathogenesis of the non-alcoholic fatty liver disease (NAFLD) [189,190]. In COVID-19 patients, serum levels of fetuin-A were found to be significantly lower compared with healthy controls. These lower levels were observed despite higher HOMA-IR, CRP, or ferritin values as required for ICU intervention. Fetuin-A deficiency was associated with a more severe COVID-19 course. This finding was correlated in many studies to the dysregulation of the glycemic control during the SARS-CoV-2 infection that decreased fetuin-A serum levels can cause, especially in diabetic pre-affected patients [134,191,192].

### 3.6. Cardiokines in COVID-19

Traditionally, the cardiomyocytes have been considered just the minimum parts of a mechanically beating vital organ at that time unrelated to the endocrine system. However, since the discovery of the production of the natriuretic peptide by those cells and the effects of these molecules on the whole body, the functions of the heart as a secretory organ have attracted attention, and the term “cardiokine” referring to the cardiac secretome has gained force. Firstly, it has been recognized that the pathophysiology of many heart disorders could begin due to a transformed cardiac hemodynamics in which the cardiac and neuroendocrinological secretome was affected by endogenous or exogenous stressors. Secondly, the roles of the cardiokines in outside-heart diseases started to be assessed. Since then, many cardiokines have been identified, and some have been studied due to their possible role in SARS-CoV-2 infection. Cardiokines can be secreted by all types of cardiac cells, such as cardiomyocytes, fibroblasts, and endothelial and vascular cells [193,194,195].

In the myocardium, cardiokines are required to maintain normal cardiac function and control pathological heart remodeling in response to injury (such as hypertension that can lead to heat hypertrophy). These molecules can also control the inflammatory stimuli in the myocardium and induce cardiac fibroblast activation to fibrosis formation and inflammation [195]. Cardiokines have also been investigated as biomarkers of MetS and as mediators of exercise-induced body regulation [196]. They were related to exerting protection against atherosclerosis formation due to the inhibition of hyperglycemia-induced endothelial cell senescence through the AMP-activated protein kinase alfa (AMPKα)/Kruppel-like factor 4 (KLF4) signaling pathway [197].

#### 3.6.1. Fibronectin Type III Domain Containing 5

The Fibronectin type III domain containing 5 (FNDC5) is a glycosylated transmembrane protein that contains a signal peptide, one hydrophobic domain inserted into the cell membrane, and two fibronectin domains. This molecule is highly expressed in skeletal muscles, cleaved, and released as the myokine irisin. However, FNDC5 is also expressed in the cardiac muscle and is secreted by the cardiomyocytes. It can alleviate OS and cardiomyocyte apoptosis via activating AKT-derived signaling pathways in animal models of doxorubicin-induced cardiotoxicity. Together with its cleaved form irisin, FNDC5 is also associated with WAT browning and, therefore, with thermogenesis. Besides these effects, FNDC5 regulates various cardiovascular diseases such as atherosclerosis, myocardial ischemia/reperfusion injury, cardiac hypertrophy, and hepatic affections such as fatty liver diseases, impaired fatty acid oxidation, and autophagy in the hepatic tissue [198,199].

Although the roles of this cardiokine during COVID-19 have not yet been assessed in clinical studies, some authors investigated its effects in laboratory models. Frühbeck et al. [200] found that FNDC5 in visceral human adipocytes reduces the SARS-CoV-2 entry points by interacting with the viral S proteins. Those authors also assessed that the FNDC5′s could also decrease S glycoprotein S1-domain-induced inflammatory visceral human adipocyte cell death due to necroptosis, pyroptosis, and apoptosis. SARS-CoV-2 has been shown to use human adipose tissue as a viral reservoir. In obese patients, the virus can trigger uncontrolled inflammatory responses that lead to various forms of cell death. These processes can worsen the prognosis of the disease. Thus, the lower the levels of FNDC5 in patients with obesity, the higher their susceptibility to SARS-CoV-2 due to the increased number of entry points and the higher the visceral adipocytes death due to inflammatory pathways.

#### 3.6.2. Growth Differentiation Factor 15

Growth differentiation factor 15 (GDF15) is a member of the anti-inflammatory TGF-β superfamily and an emerging mediator of immune responses and tissue tolerance to inflammation through metabolic adaptation [201,202]. Induced by stress, it is the product of a gene on the human chromosome 19p13.11-13.2 and synthesized as pro-GDF15. Further, it is dimerized, cleaved, and secreted as mature GDF15, being considered an essential regulator of appetite through the glial-derived neurotrophic factor receptor alpha-like (GFRAL) and present at high levels in comorbidities such as obesity and diabetes [203,204]. In mouse models, GDF15 acts as a regulator of obesity, decreasing glucose intolerance and increasing lipid metabolism [205].

GDF15 binds to the GDNF receptor family member GFRAL in the hindbrain to act on metabolic effects, appetite reduction, and body weight via the neurotransmitter cholecystokinin (CCK). In experimental studies with mouse models, GDF15 has been shown to increase fatty acid oxidation and glucose uptake and reduce oxidative stress and inflammation by activating the PPARβ/δ-AMPK-p53 pathway. In addition, GDF15 has been shown to contribute to the increase in the levels of the peroxisome proliferator-activated receptor (PGC)-1α and lipin-1 coactivator by activating PPARβ/δ [206]. Furthermore, as a member of the TGF-β family, peripheral effects may be associated with receptors of the TGF-β/Smad signaling pathway, such as ALK-5/TGF-βRII, TGF-βRI, TGF-βRII, and the epithelial growth factor receptor ErbB2 [207,208,209].

Under physiological conditions, GDF15 is expressed by skeletal and smooth muscle cells, macrophages, adipocytes, and cardiomyocytes [210]. Released by various cell types during acute and chronic inflammation, elevated levels of GDF15 are also reported in the elderly, in late pregnancy, and with high-intensity exercise. It is considered a marker of mortality in the elderly and has been established as a predictor of the severity of bacterial and viral infections [211]. In viral infections, GDF15 acts by attenuating antiviral immune responses, significantly affecting the outcome of the disease [212]. In mouse models, GDF15 was overexpressed in acute exacerbations of chronic obstructive pulmonary disease (COPD) caused by human rhinovirus (RV), in addition to promoting virus replication and increasing the inflammatory state, which is possibly explained by impaired production of interferon-γ1 (IFN-γ1) [212].

GDF15 has emerged as an important marker of severity in complications caused by SARS-CoV-2 due to its correlation with hypoxia, tissue damage, and aging [213]. Studies have shown that GDF15 is related to inflammatory cytokines, such as IL-6 and CRP, suggesting an essential role in maintaining inflammation throughout viral infections [202,214]. In COVID-19, the cytokine storm contributes to rapid deterioration in patients with pre-existing chronic inflammatory conditions such as obesity, hypertension, and diabetes, which are correlated with elevated levels of GDF15 [215].

### 3.7. Studies Evaluating the Role of Organokines in COVID-19 Patients

#### 3.7.1. Miscellaneous

Some clinical trials showed the roles of organokines in COVID-19 infection. Tonon et al. [90] conducted a retrospective observational case-control study to evaluate the discriminatory ability of adipokines to identify COVID-19-related pneumonia and assess disease severity. The results showed that decreases in the adiponectin/leptin ratio were significantly associated with AT dysfunction and COVID-19-related pneumonia onset in COVID-19 patients. However, this study has a small sample size.

Di Filippo et al. [17] conducted a single-center, prospective observational study to evaluate whether adiponectin and leptin serum levels could be associated with the inflammatory burden during COVID-19 infection. The results showed that increased adiponectin and reduced leptin or the rise in adiponectin/leptin ratio could be related to the inflammatory burden in COVID-19 and its mortality, principally in pre-existing cardiometabolic diseased patients. However, this study had some limitations. Firstly, the sample size was relatively small in each subgroup, which decreased the significance of between-group comparisons. The adiponectin and leptin serum levels were only evaluated upon patients’ admission, which did not allow the researchers to draw conclusions based on the causal correlations between the adiponectin/leptin ratio and the possible clinical outcomes in the studied COVID-19 sample.

Minuzzi et al. [149], in a prospective study conducted with hospitalized COVID-19 patients, tried to assess whether age, sex, or adiposity (obesity) could influence the AT endocrine response during the infection. The results showed that neither adiponectin nor leptin was associated with worsened outcomes among the diseased. However, the BDNF/adiponectin ratio was significantly associated with the worst prognosis during the infection. The study presented a new approach to managing the healthcare of COVID-19 patients. However, a significant limitation of the study was that the authors did not match the non-obese and obese groups for the most prevalent cardiometabolic risk factors, namely, hypertension and dyslipidemia. These risk factors are also associated with adipose tissue dysfunction and could have influenced the expression of BDNF, adiponectin, and leptin even before the onset of COVID-19 in the studied patients. The two subgroups had no significant differences concerning sex, age, and medication use.

In a single-center, prospective observational study, Blot et al. [111] evaluated the relationship between COVID-19-related severe pneumonia and adipokines, immunological statuses, and COVID-19 outcomes. The results showed that only leptin serum levels negatively correlated with IL-6 and IL-1β serum levels. Nonetheless, this study had limitations such as a small sample size, and the predictive and demographic characteristics between the two subgroups were not matched, principally in the case of the comorbidities distribution.

In a cross-sectional study with COVID-19 patients, Michał Kukla et al. [134] evaluated whether fetuin-A, irisin, FGF-21, and PTX3 serum levels could be associated with the development and progression of the SARS-CoV-2 infection. The results showed that those patients with COVID-19 had decreased levels of fetuin-A compared with healthy controls and that these decreased levels were associated with more severe disease courses. Obese patients with COVID-19 also had increased levels of FGF-21 compared with overweighted ones, especially those diagnosed with MetS. PTX3 was higher in those COVID-19 patients that possessed higher HOMA-IR values. Additionally, upregulated PTX3 was considered a predictor of COVID-19 severity. However, this study had some limitations. Firstly, the sample size comprised a relatively small number of patients, especially in the control group. Secondly, this study evaluated hepatokines in a cross-sectional way, and FGF-21 to be studied longitudinally in this sample also included obese patients.

#### 3.7.2. PTX3

In a retrospective cross-sectional study, Assandri et al. [216] assessed whether PTX3 serum concentrations could be associated with COVID-19 severity and its performance in detecting the disease prognosis without measuring other biochemical parameters. These authors comprised data from COVID-19 patients. The results showed that PTX3 levels were higher among ICU patients compared with non-ICU. Additionally, receiver operator characteristic (ROC) curves demonstrated that PTX3 had higher accuracy than CRP, lactate dehydrogenase (LD), or ferritin in identifying ICU necessity during COVID-19. By univariate and multivariate logistic regression analysis, the results also demonstrated that PTX3 was the only significant proteomic predictor of ICU-related COVID-19 stay necessity among the diseased individuals after controlling their pre-existing comorbidities. Nevertheless, this study had some limitations. The sample size was relatively small, and the study’s logistic regression did not have a validation cohort.

Hansen et al. [217], in a prospective cross-sectional study conducted with COVID-19 patients, evaluated whether PTX3 could be a valuable plasma biomarker to assess COVID-19 severity and prognosis. The sample of COVID-19 patients comprised survivors and non-survivors, and the results showed that among non-survivors, PTX3 serum concentrations were significantly higher. After adjustment for covariates, the odds of 30-day mortality increased 2-fold for each doubling of the serum PTX3 levels. Additionally, PTX3 serum levels significantly predicted 30-day COVID-19 patients’ respiratory failure and their respective mortality risks. Although this study had strong results compared with a validation cohort, it also had one limitation that must be addressed. Notably, this study’s derivation and validation cohorts had significant differences between age and sex distribution and in the availability of covariates and pharmacological treatment utilized to treat COVID-19-diseased patients.

Genç et al. [182], in a cross-sectional study that comprised COVID-19 patients, including survivors and non-survivors, aimed to evaluate whether PTX3 serum concentrations could predict the disease’s prognosis. The results showed that PTX3 levels were significantly higher in the non-survivor patients than in survivors. The authors also performed a receiver operating characteristic curve analysis and identified a cut-off point value of 3.73 for PTX3 as a predictor of mortality in all patients. This value had a sensitivity and specificity of 65%. As a limitation of this study, we can cite the relatively small sample size and that the authors only investigated the PTX3 serum levels on the day of the patient’s hospital admission. This study did not have a control group for comparisons, which decreased its generalizability.

Gutmann et al. [181] conducted a prospective study to evaluate PTX3 serum levels in 123 hospitalized COVID-19, 25 non-COVID-19 ICU sepsis, and 30 healthy control patients. Machine learning analysis identified “Age, RNAemia” and “Age, PTX3” as the top binary signatures associated with 28-day ICU mortality in COVID-19 patients. Longitudinal comparisons revealed a distinct proteomic trajectory related to mortality in COVID-19 ICU patients, with the recovery of several liver-derived proteins indicating survival.

Tong et al. [184], in a retrospective study comprising COVID-19 patients, evaluated whether PTX3 serum concentrations could be associated with disease-related coagulopathies. The results showed that in those patients that presented D-dimmer levels ≥ 1 mg/L, PTX3 levels significantly and positively correlated with the occurrence of coagulopathies and the COVID-19 disease severity. The serum concentrations of PTX3 also significantly and positively correlated with the serum levels of D-dimmer. Nevertheless, this study had some limitations, such as the small sample size and the absence of a control group. Secondly, the researchers did not measure the natural coagulation system proteins or even endothelial dysfunction biomarkers, so the specific coagulation disturbances associated with PTX3 and SARS-CoV-2 in this cohort of patients were not assessed.

Kerget et al. [185] conducted a cross-sectional study with a sample of COVID-19 patients to investigate the relationships between PTX3 gene polymorphisms (rs2305619 (281A/G) and rs1840680 (1449A/G)) and the development of macrophage activation syndrome (MAS) during the active disease. Of those patients, 46 were admitted to the ICU due to MAS, and 48 were without MAS or ADRS. The results showed that PTX3 281A/G allele and genotype frequencies did not deviate from the Hardy–Weinberg (HW) equilibrium between the MAS and non-MAS groups. In turn, the PTX3 1449A/G allele and genotype frequencies significantly deviated from the HW equilibrium in the non-MAS group but did not deviate from the MAS group at the same significance. Additionally, the PTX3 1449A/G polymorphism showed that GG genotype individuals had higher serum levels of PTX3 than those with the AA and AG genotypes. However, the AG genotype was significantly more frequent among the non-MAS group individuals, and the AA genotype was significantly more frequent in the MAS group. Lastly, the analyses showed that those patients with the AG genotype of the PTX3 gene 1449A/G polymorphism were relatively more protected from MAS than those with the AA genotype. This study was dense and had many strengths, principally showing that polymorphism differences can act differently in the PTX3 hepatokine effects in COVID-19 infection. Although the sample comprised a relatively good number of patients, the authors limited the analyses of the PTX3 1449A/G and 281A/G polymorphisms for a single race, which highly decreases the study’s generalizability and can create statistical deviation.

Gritti et al. [186] conducted a prospective, observational cross-sectional study with hospitalized COVID-19 patients to evaluate whether siltuximab could be used as treatment against COVID-19 infection. The results showed improvements in the respiratory statutes and survival of the treated patients and reductions in IL-8 and PTX3 serum concentrations. Some limitations of this study are that it was not randomized and the sample size was small.

In a cross-sectional study, Moulana et al. [217] investigated in a sample of COVID-19 patients the associations between PTX3 serum concentrations and COVID-19 disease severity. The COVID-19 selection comprised ICU-admitted and non-ICU patients and healthy controls. The results showed that the ICU and non-ICU COVID-19 individuals had significantly higher PTX3 levels than the controls and that PTX3 was higher among ICU-admitted patients in contrast with non-ICU but without significance. The results also demonstrated that PTX3 and CRP levels were significantly associated with the disease‘s prognosis and severity definition.

#### 3.7.3. PGRN

Özgeris et al. [125] conducted a pilot cohort study to evaluate whether PGNR serum levels could be associated with COVID-19 prognosis and predict disease severity course. The results showed that the serum PGRN levels were significantly higher among the COVID-19 patients compared with healthy controls. The ROC curve analysis identified PGRN as a better biomarker for disease prognosis than CRP (0.931 ± 0.08). However, this study presented a small sample size.

Yao et al. [126] conducted a cross-sectional cohort study with a sample of COVID-19 and healthy control patients to establish quantitatively whether the serum levels of PGRN and sVCAM-1, sICAM-1 (intercellular adhesion molecule-1, sP-selectin, and sE-selectin) would be elevated in the infected patients when compared with the controls. Additionally, the authors wanted to evaluate the possible associations between PGRN levels and endothelial activation markers. As a result, it was encountered that COVID-19 patients had significantly higher serum levels of PGRN and sVCAM-1 in comparison with healthy controls. Although these results favor the authors to consider PGRN serum levels as an endothelial activator during COVID-19, this study had some limitations, such as a small sample size, patients enrolled were very heterogenous, and most patients had mild/moderate COVID-19 infections, which do not encompass the complete picture of the disease. In addition, the impact of possible co-infections with other microorganisms on the PGRN and the adhesion molecules’ serum levels was not considered in the study’s analyses.

Brandes et al. [218], in a comparative, observational study, evaluated sepsis, community-acquired pneumonia (CAP), systemic inflammatory response syndrome (SIRS), and COVID-19 patients to investigate the relationships between PGRN plasma levels between severely ill patients in comparison with healthy controls. The results showed that PGRN serum levels differed significantly between sepsis, CAP, SIRS, and COVID-19 patients, possibly representing PGRN as a novel indicator for the differentiation between those four different disorders. However, the sample size was small and in clinical reality the sepsis prevalence in patients with distracting conditions, such as SIRS, CAP, and COVID-19, is generally lower than in this study’s investigations.

Rieder et al. [219] conducted a prospective, single-center study to evaluate whether changes in serum PGRN levels could be associated with COVID-19. The sample comprised COVID-19 and healthy controls, and the results demonstrated that PGRN was significantly and explicitly upregulated in COVID-19 patients compared with healthy controls. Possible limitations of this study were the small sample size and the grater heterogenicity between the studied groups.

#### 3.7.4. OPN

Reisner et al. [162] conducted a pilot study where they examined the correlation between OPN levels and the severity and clinical presentation of COVID-19. The study found that asymptomatic or minimally symptomatic children had median OPN levels of 76.72 ng/mL, while patients with mild/moderate symptoms had median OPN levels of 196.79 ng/mL. Patients with severe symptoms had higher median OPN levels of 430.31 ng/mL, while patients diagnosed with multisystem inflammatory syndrome (SIM-C) had the highest median OPN levels of 598.11 ng/mL. These results demonstrate that OPN may be a more sensitive marker of disease severity than other features analyzed in the study, such as CRP and ferritin, maximum erythrocyte sedimentation rate, soluble interleukin 2R, and interleukin 6, which did not show differences significantly between the studied groups. On the other hand, Varim et al. [161], in a prospective cohort study with hospitalized patients, found OPN values of 13.75 ng/mL (11.30–17.07 ng/mL) in hospitalized COVID-19 adult patients in critical condition and 9.85 ng/mL (8.32–19.23 ng/mL) in non-critical patients. These authors also described that OPN could be used as a marker to predict the severity of the COVID-19 disease.

Hayek et al. [169], in a retrospective cohort study with hospitalized patients with COVID-19, demonstrated that infected patients had higher serum levels (96.63 ng/mL) of OPN compared with healthy controls (16.56 ng/mL). In addition, OPN levels ≥ 140.66 ng/mL were associated with a 4.9-fold increase in the chance of mechanical ventilation and a 3.5-fold increase in the possibility of death. The authors also observed significant correlations between routine biomarkers such as procalcitonin, ferritin, CRP, and D-dimers with serum OPN levels. Furthermore, OPN levels in the third tertile of the multivariable binary logistic regression analysis identified OPN as an independent predictor of death. These data support the ability of OPN to work as a biomarker for prognosis and identification of severity in patients with COVID-19.

Fonseca et al. [220], while investigating how SARS-CoV-2 infection and inflammation systematically affect older African-American men, demonstrated that cytokines such as IL-18, IL-6, IL-33, MIP-1α, and OPN were elevated in COVID-19 patients hospitalized in the ICU compared with the control group. OPN serum levels were also elevated in non-survivors and patients examined on dialysis. In the same perspective, Bai et al. [221] conducted a retrospective cohort study to evaluate OPN and Gal-9 in COVID-19 patients. They looked at the relationship between these markers and the severity of pneumonia caused by COVID-19. ROC analysis found that the cleaved forms of OPN (FL-OPN, full-length osteopontin; Ud-OPN, undefined osteopontin) had high power in discriminating severity between two patient groups. The first group consisted of COVID-19 patients with pneumonia, while the second group had mild clinical symptoms. The researchers found that the pneumonia severity was significantly higher in the first group. On treatment with Tocilizumab, a humanized monoclonal antibody that recognizes the soluble IL-6 and membrane-bound IL-6 receptor, Ud-OPN levels decreased significantly. Thus, cleaved OPN forms may also open perspectives for their use in assessing the severity of COVID-19 pneumonia.

#### 3.7.5. Adiponectin

Kearns et al. [91] conducted a retrospective cross-sectional study to evaluate whether adiponectin levels could be associated with respiratory failure in COVID-19 patients. To decrease biases, the authors compared COVID-19 respiratory failure with non-COVID-19 respiratory failure individuals (8 with bacterial pneumonia and 1 with hospital-acquired pneumonia, with healthcare-associated pneumonia, and with *Pseudomonas aeruginosa*, 4 with influenza, and 1 with parainfluenza and with metapneumovirus). The results showed that adiponectin serum levels were significantly lower during COVID-19 respiratory failure, even after adjustments for age, sex, pre-existing diabetes mellitus, BMI, type of enteral nutrition, or glucocorticoid therapy use. This study has strengths because serial measurements of adiponectin were made for over 72 h. However, some limitations should also be listed. This study had a small sample size considering that only 12 COVID-19 patients were analyzed. Additionally, no demographic and prognostic characteristics matched between the studied cohorts, which can decrease the study’s generalization.

In a retrospective case-control study, Reiterer et al. [88] evaluated whether patients with and without ARDS adiponectin levels could be associated with hyperglycemic-derived adverse severe outcomes in COVID-19 patients. The results showed that in this cohort, patients with COVID-19 presented lower levels of adiponectin compared with controls and that these levels were significantly associated with the occurrence of hyperglycemic-derived adverse severe outcomes during disease progression. However, this study had some limitations. Firstly, the sample was not matched in demographic and prognostic characteristics, pre-existing comorbidities, or in-hospital treatments. Secondly, the patients were not fasted to assess blood glucose levels, being hyperglycemia predominantly characterized by beta cell failure and HOMA-IR assays. Lastly, glucocorticoid treatments and the eminence of septic shock, which are events that alter blood glucose, appeared to be not addressed for the hyperglycemic assessments during this study.

Caterino et al. [92] conducted a single-center cohort, cross-sectional study with COVID-19 patients to assess whether adiponectin levels could be associated with the inflammatory burden and lipidomic profile during COVID-19. The results showed that serum adiponectin levels significantly and positively correlated with the IL-6, ceramides, and glycerophospholipids levels among the diseased individuals. Besides these results, this study had some limitations. Firstly, this study did not have a control cohort. Secondly, the predictive and demographic characteristics were not matched between the subgroups insofar as the severe patients were much older than the mild and the moderate individuals, which can decrease the study’s generalizability. The severe patients also had comorbidities that could alter their lipid profiles, such as obesity.

#### 3.7.6. Leptin

Larsson et al. [110], in a single-center, cross-sectional cohort study, evaluated whether leptin levels could be associated with mortality rates or ICU length stay during COVID-19 in severely infected patients. The results showed that leptin serum levels were significantly higher in the COVID-19 patients than in healthy controls, with more prominent values in the female population. However, leptin did not correlate with this cohort’s mortality rates of ICU length stay. Although this study presented a good sample size and a control group of healthy individuals, it had limitations. The data from the sample were collected over two years. During this time, strategies provided to COVID-19 patients evolved, and many people were also vaccinated against SARS-CoV-2. These facts certainly caused heterogeneities in how COVID-19 patients would appear in the ICU admission; therefore, leptin levels could also be the collected data.

Wang et al. [109] conducted a single-center, cross-sectional cohort study to evaluate whether leptin serum levels could be associated with pro-inflammatory cytokines secretion profile and disease progression during severe and mild COVID-19 infections. The sample comprised mild and severe COVID-19 patients, and the results of the infected individuals were compared with healthy controls. Leptin showed significant power in predicting disease progression and severity and significantly correlated with decreased lymphocyte counts in the studied sample. Notably, leptin serum levels were associated with M1 macrophage polarization in the cohort of severe patients. Although these results can be considered promising, some limitations of this study must be cited. First of all, the sample size can be regarded as small for this research. In addition, data on cytokine secretion were only available from three mild and three severe cases, which decreases the study’s generalizability. Moreover, not all patients had all flow cytometry analyses conducted due to late hospital admission.

During autopsy studies of COVID-19 patients, Santos et al. [112] discussed the genetic associations between leptin and the inflammatory burden during the SARS-CoV-2 infection. These authors divided the autopsied patients into hypertension, hypertension + T2DM, and hypertension + T2DM + obesity subgroups, and all the findings were compared with healthy controls. The results showed that the leptin gene was overexpressed among the examined obese patients and its related genes, LCK and EGR2, which theoretically can demonstrate pulmonary damage through the activation of inflammation and monocyte dysregulation, respectively. Additionally, it is essential to mention that the sample size in the study was small. Besides that, there were no obese patients in the control group. Lastly, the type of drugs used by the dead patients during hospitalization by COVID-19 and other confounding factors could have masked the genetic evaluations by interfering directly with the lung cells.

In a cross-sectional study, van der Voort et al. [114] evaluated in a cohort of SARS-CoV-2 ventilated patients how leptin serum levels could be associated with SARS-CoV-2 infection in comparison with critically ill non-infected controls. The results showed that leptin levels were significantly higher in the studied COVID-19 patients than in the controls. The limitations of this study include the sample size and the controls did not have other specific respiratory conditions except for various affections, such as sepsis, cardiogenic shock, intracerebral hemorrhage with respiratory failure, a postoperative condition due to aortic valve replacement, and polytrauma.

#### 3.7.7. GDF15

In a retrospective observational case-control study performed by Teng et al. [213], potential biomarkers were evaluated in patients categorized according to the severity of COVID-19 infection. In the study, only 11 factors were associated with disease severity, including GDF15, transferrin, TNF-R1-related apoptosis-inducing ligand (TRAIL R1), VCAM-1, secreted frizzled-related proteins-3 (sFRP-3), fatty-acid binding protein-2 (FABP2), insulin-like growth factor binding protein-1 (IGFBP-1), IGFBP-4, differentiation group 200 (CD200), IL-1F7, and IL-5Rα. When analyzing the serum levels of GDF15, a proportional increase was observed with the severity of the COVID-19 infection, recording levels of 305.930 ± 85.8 pg/mL in the group classified as moderate, 422.5 ± 80.9 pg/mL in the severe group, and 621 pg/mL in the critical group. In addition, GDF15 levels observed a linear reduction in patients discharged from the hospital and an increase in worsening symptoms before death. This evidence may relate to GDF15 as a probable indicator of disease severity in patients infected with COVID-19.

In a case series, Guadiana et al. [222], while evaluating the potential role of GDF15 in predicting in-hospital mortality during COVID-19, observed that serum levels of this biomarker increased in patients who died during the hospital stay. Average GDF15 levels of 2590 ng/L (1886–4811 ng/L) were recorded for the survivor patients, and 9448 ng/L (6462–17,707 ng/L) were recorded for the non-survivors. In this study, despite the small sample size and non-performance of multivariate analysis, GDF15 showed good discrimination capacity, proposing a potential biomarker in evaluating prognosis in patients with COVID-19. In the same perspective, the prospective study by Alserawan et al. [223], which analyzed patients with COVID-19, was able to show that patients with low levels of the saturated oxygen/fraction of inspired oxygen (SpO2/FiO2) ≤ 400 had higher levels of GDF15, IL-6, D-dimer, and CRP. In addition, ROC curve analyses identified that GDF-15 ≥ 1675 pg/mL was associated with worse respiratory function.

Notz et al. [202], in a retrospective single-center cohort study, evaluated the roles of GDF15 in the immune response during COVID-19 in patients with COVID-19-induced ARDS. COVID-19 patients, of which 9 were survivors and 4 died from multi-organ failure after a median hospitalization length stay of 14 days, were analyzed. The results demonstrated that patients with COVID-19 presented elevated levels of IL-6, lymphocytopenia, and delayed cytotoxic immune defense. Moreover, the IL-10 and GDF-15 levels were also elevated. However, this study lacks non-COVID-control patients and has small sample size limitations.

In a prospective observational study carried out by Myhre et al. [214] to investigate the associations of GDF15 in patients hospitalized for COVID-19, it was observed that in patients admitted to the ICU or who died, GDF15 levels up to the 3rd day of hospitalization increased by an average of 1208 pg/mL, while survivors not admitted to the ICU had an average reduction of 86 pg/mL. This scenario changed when the length of stay was extended to nine days, when GFG15 continued to increase, reaching a median of 8031 pg/mL (3589–16,003 pg/mL) in ICU patients. Furthermore, elevated levels of GDF15 have been linked to SARS-CoV-2 viremia, suggesting a relationship between the cytopathic effects of the virus and GDF15 expression. Another finding was the inverse association between GDF15 levels and oxygen saturation, with higher median concentrations (4049 pg/mL) in patients with hypoxemia (SpO2 ≤ 90%) than in patients without hypoxemia (2331 pg/mL), suggesting the role of this biomarker in the identification of silent hypoxemic patients.

#### 3.7.8. Myostatin

He et al. [137], in a retrospective cohort study, evaluated in a sample of COVID-19 patients whether some metabolomic factors could be involved in the regulation of myostatin expression during COVID-19 infections and whether this myokine could be associated with the ordinary IR and lipid dysregulations of the disease. The results showed that the SARS-CoV-2 pathophysiological pathways increased the expression of RE1-silencing transcription factors. The REST factors modulated the presentation of the secreted myostatin at the transcriptional levels, which resulted in glucose and lipid metabolism disturbances.

#### 3.7.9. BDNF

Asgarzadeh et al. [150], in a prospective cohort study, evaluated whether BDNF serum levels could be associated with COVID-19 manifestations, especially neurological impairments and infection-induced hypoxia. The sample comprised COVID-19 patients; some had neurological COVID-19-related manifestations, and others did not. The results showed that COVID-19 patients had significantly lower BDNF serum levels than healthy controls. Furthermore, BDNF levels were even lower among those patients with COVID-19-related neurological manifestations and higher in those without neurological impairments but with high fever and dyspnea. BDNF also presented a negative association with oxygen-therapy necessity. These results suggest an association between dysregulated BDNF serum levels and hypoxia in promoting COVID-19 manifestations, particularly neurological ones.

Petrella et al. [151] conducted a pilot cohort study to identify BDNF changes in the serum of Italian adolescents that contracted COVID-19 during the second wave of the disease. The results showed that BDNF serum levels were higher in only post-infected-COVID-19 symptomatic and future long-COVID-19 girls than healthy controls. Additionally, BDNF levels were unchanged in asymptomatic individuals compared with the control group. However, this study presented a minimal sample size, with each studied group containing ten adolescents.

**Figure 5:**
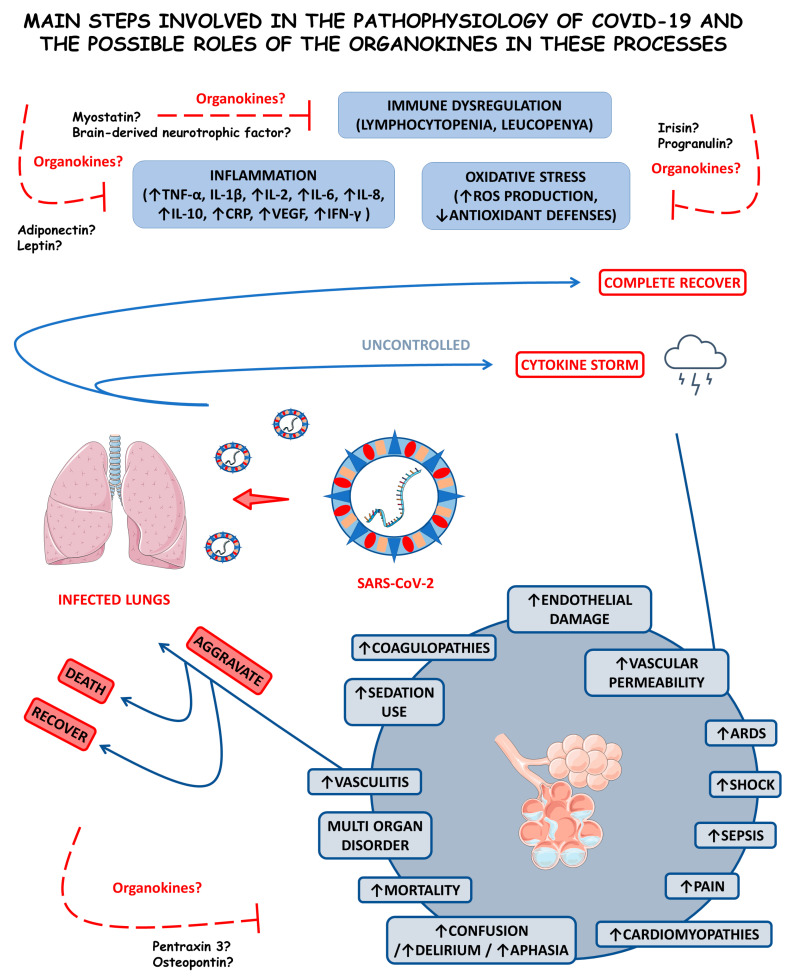
Main steps involved in the pathophysiology of COVID-19 and the possible roles of the organokines in these processes. Inflammation, immune dysregulation, and oxidative stress are part of the infection caused by SARS-CoV-2. Together, they can drive acute respiratory distress syndrome (ARDS) and multi-organ disorders. Organokines can play a role in the occurrence of these phenomena. Understanding how organokines may be involved in developing COVID-19 will certainly contribute to developing new and modern diagnostic and therapeutic strategies for healthcare people infected by SARS-CoV-2. ↑, increase; ↓, decrease; TNF-α, factor tumor necrosis alfa; IL-1β, interleukin 1 beta; IL-2, interleukin 2; IL-6, interleukin 6; IL-8, interleukin 8; IL-10, interleukin 10; CRP, c reactive protein; VEGF, vascular endothelial growth factor; IFN-γ, interferon gamma; and ROS, reactive oxygen species.

Figure 5 shows the main steps involved in the pathophysiology of COVID-19 and interrogates the roles of organokines in these processes.

## 4. Conclusions

The emergency of COVID-19 caused a pandemic responsible for millions of infected people and deaths worldwide. Since it is a new infection without specific treatments and guidelines based on scientific evidence, specialists had and still have difficulties dealing with this condition.

Considering this scenario and the fact that organokines are increasingly recognized as critical mediators of inflammatory disease development and control, it is essential to associate them with the effects of the virus in the organism, which are triggered mainly by the cytokine storm. These molecules produced by different organs have the potential to be used as adjuvant biomarkers to predict the severity of the illness and severe outcomes to characterize important disease manifestations. However, much research still needs to be conducted to transpose and incorporate these data into clinical practice as interventions and therapeutic options against COVID-19.

## Data Availability

Not applicable.
